# Isolated Talar Metastasis of Renal Cell Carcinoma Treated With Tibiocalcaneal Arthrodesis Using an Ilizarov External Fixation

**DOI:** 10.7759/cureus.77275

**Published:** 2025-01-11

**Authors:** Shuichi Chida, Koji Nozaka, Hiroyuki Tsuchie, Hiroyuki Nagasawa, Naohisa Miyakoshi

**Affiliations:** 1 Orthopedic Surgery, Hiraka General Hospital, Akita, JPN; 2 Orthopedic Surgery, Akita University Graduate School of Medicine, Akita, JPN

**Keywords:** acrometastasis, ilizarov external fixation, talar metastasis, tibiocalcaneal arthrodesis, wide resection

## Abstract

Acrometastases to the feet are exceedingly rare. We report a case of isolated talar metastasis from renal cell carcinoma successfully managed through surgical excision and reconstruction with Ilizarov external fixation. A 65-year-old male presented with a six-month history of right ankle pain. Radiographs revealed an osteolytic lesion in the talus. An incisional biopsy confirmed the diagnosis of metastatic renal cell carcinoma. Systemic evaluation showed no recurrence in the right kidney or metastases beyond the right talus. En bloc resection of the talus was performed via an anterior approach, followed by tibiocalcaneal arthrodesis using Ilizarov external fixation. At the four-year follow-up, the patient remained pain-free while walking and showed no evidence of recurrence or additional metastases.

## Introduction

Acrometastases to the feet are extremely rare. The most common primary malignancies associated with distal skeletal metastases originate from the lungs, breasts, or gastrointestinal tract [1,2]. We report a unique case of isolated talar metastasis from renal cell carcinoma that was successfully managed through surgical excision followed by reconstruction using Ilizarov external fixation.

## Case presentation

**Figure 1:**
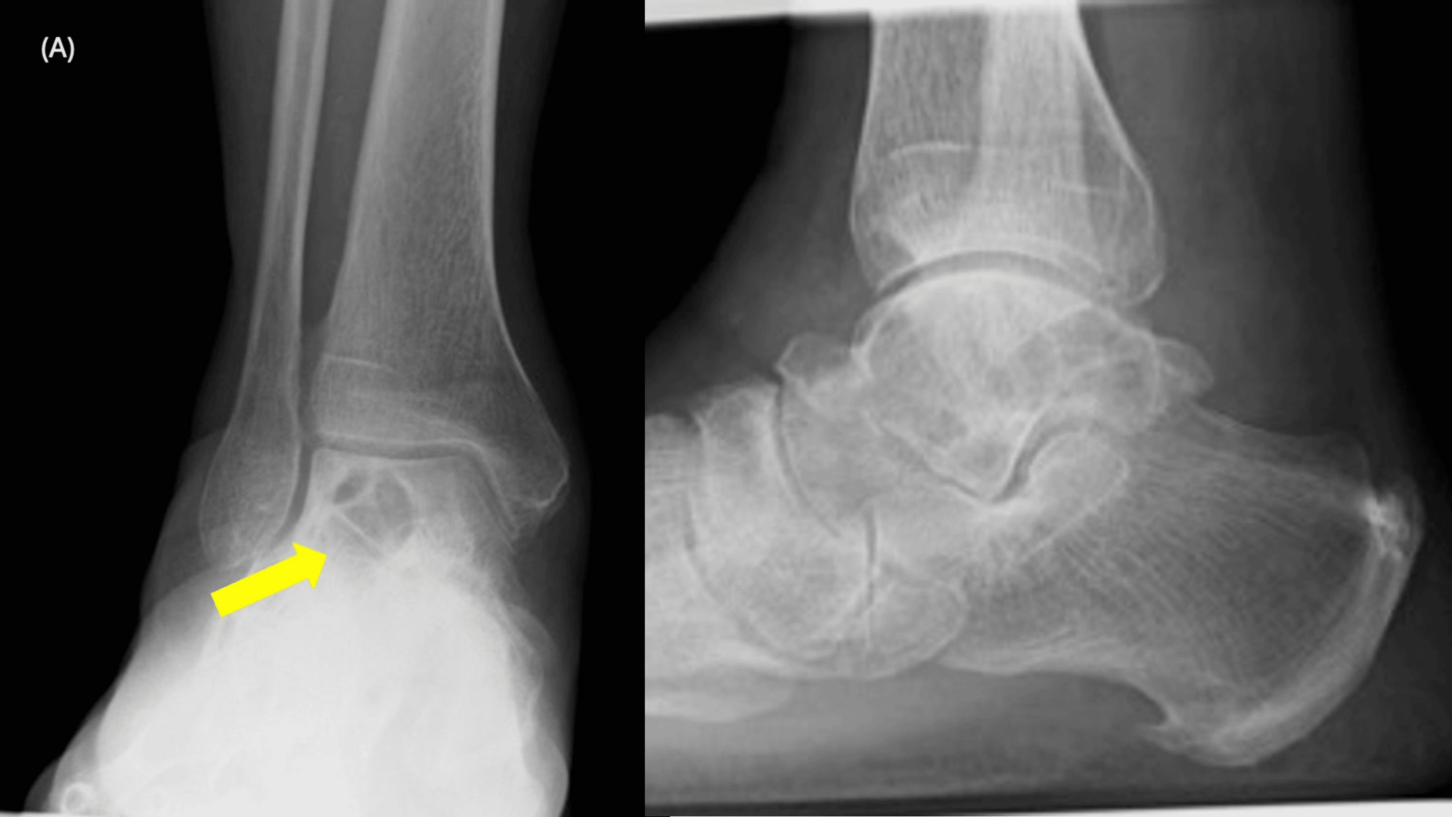
Preoperative radiographs indicating osteolytic lesion in the talus (yellow arrow)

**Figure 2:**
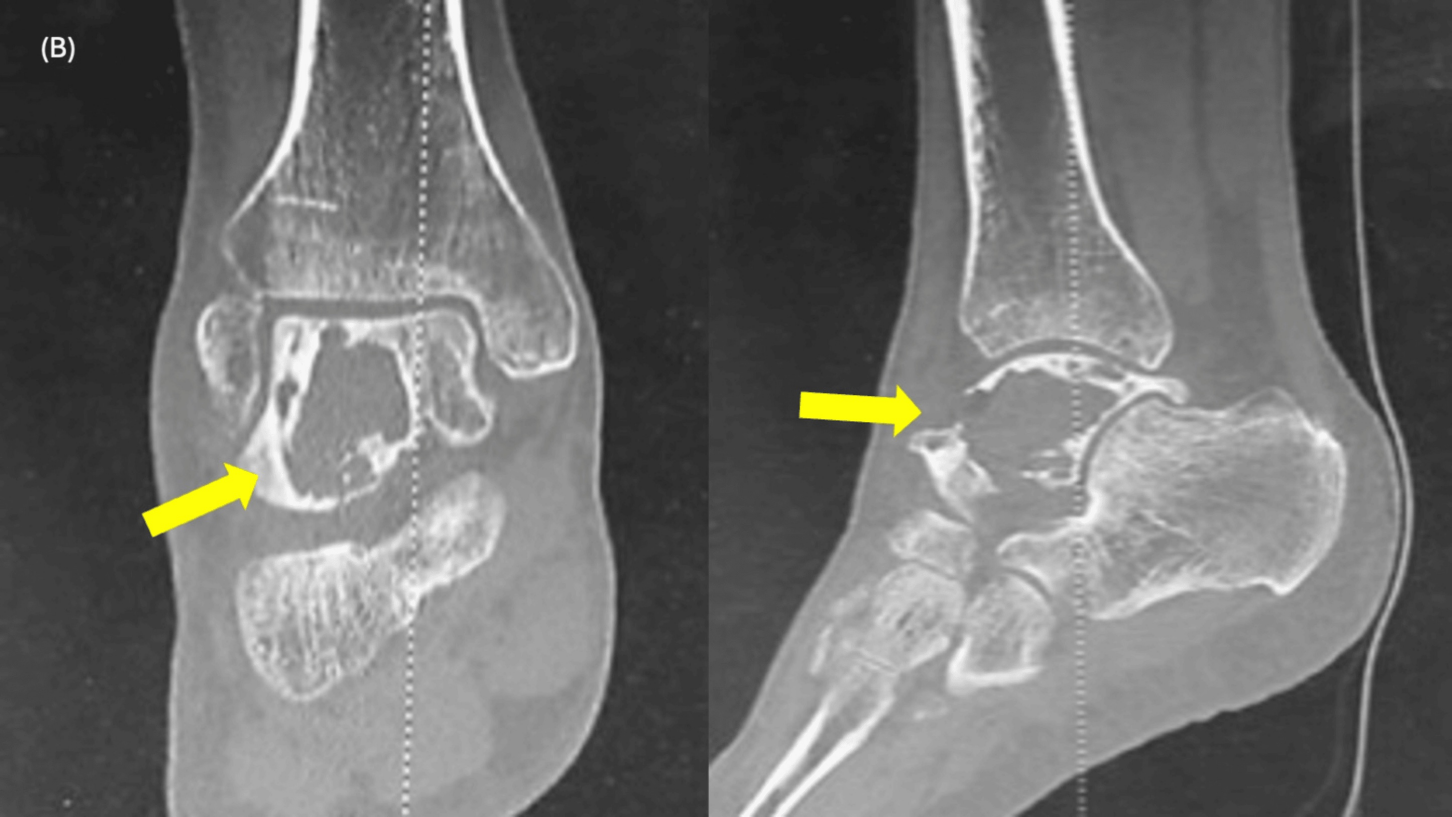
CT showing osteolytic lesion in the talus (yellow arrows) CT: computed tomography

**Figure 3:**
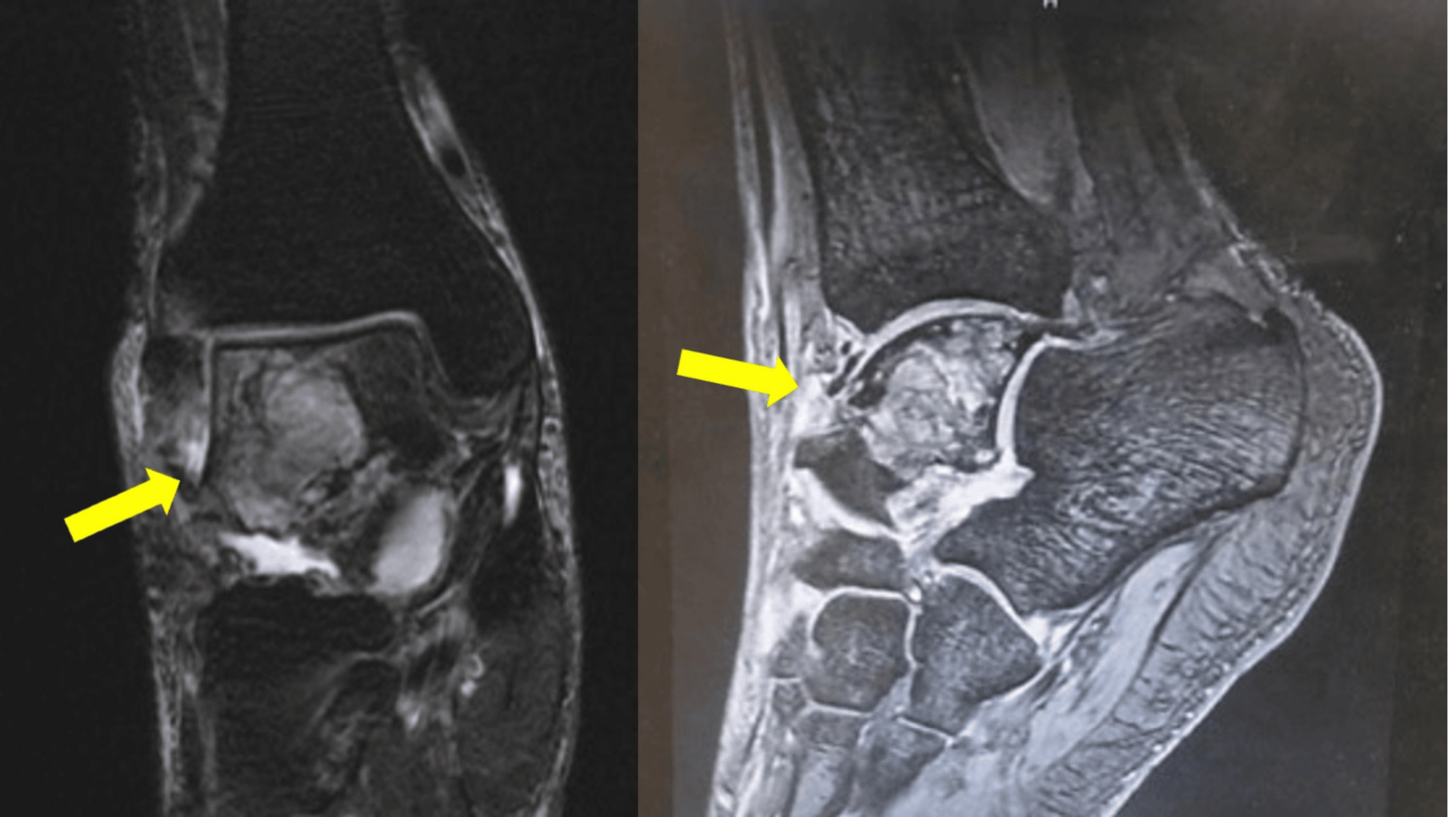
Enhanced MRI MRI confirmed the presence of an interosseous osteolytic lesion in the talus (yellow arrows) MRI: magnetic resonance imaging

**Figure 4:**
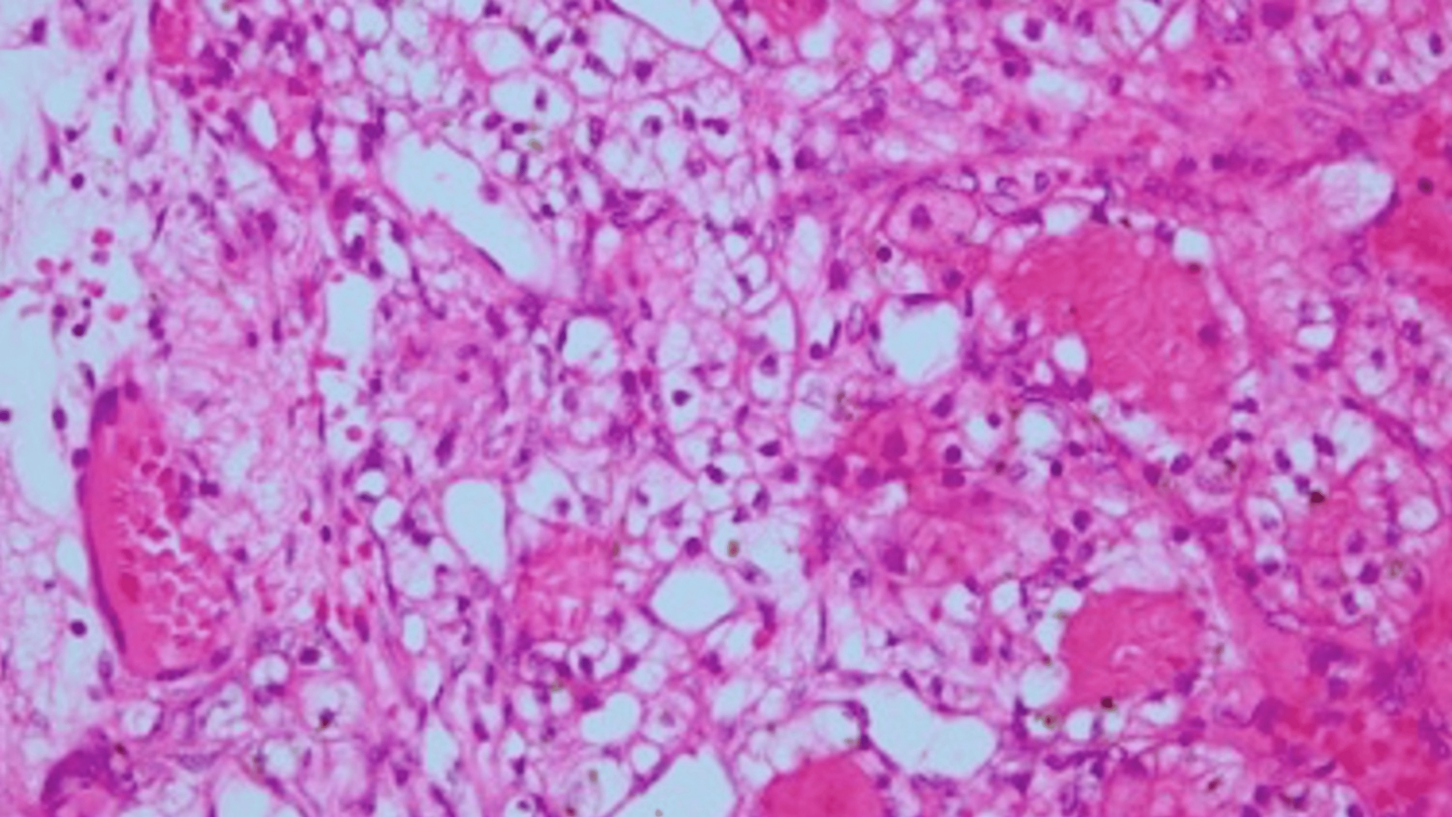
Histopathological photo of an incisional biopsy The biopsy confirmed that the lesion originated from renal cell carcinoma (magnification: x100; hematoxylin and eosin stain)

A 65-year-old Japanese male had presented to a local physician with a six-month history of right ankle pain and had been initially treated with nonsteroidal anti-inflammatory drugs. However, as the pain progressively worsened with movement, he had sought further evaluation at another institution. His medical history had been notable for right renal cell carcinoma treated 14 years earlier. Radiographs and CT had revealed an osteolytic lesion in the talus (Figures 1-2). MRI had confirmed the presence of an interosseous osteolytic lesion in the talus, with no involvement of the ankle joint or surrounding soft tissue. The lesion had demonstrated low signal intensity on T1-weighted imaging and high signal intensity on T2-weighted and enhanced MRI (Figure 3). An incisional biopsy performed had led to a diagnosis of renal cell carcinoma metastasis (Figure 4). The patient had been subsequently referred to our institution. A systemic evaluation revealed no recurrence in the right kidney and no metastasis to other sites beyond the right talus.

**Figure 5:**
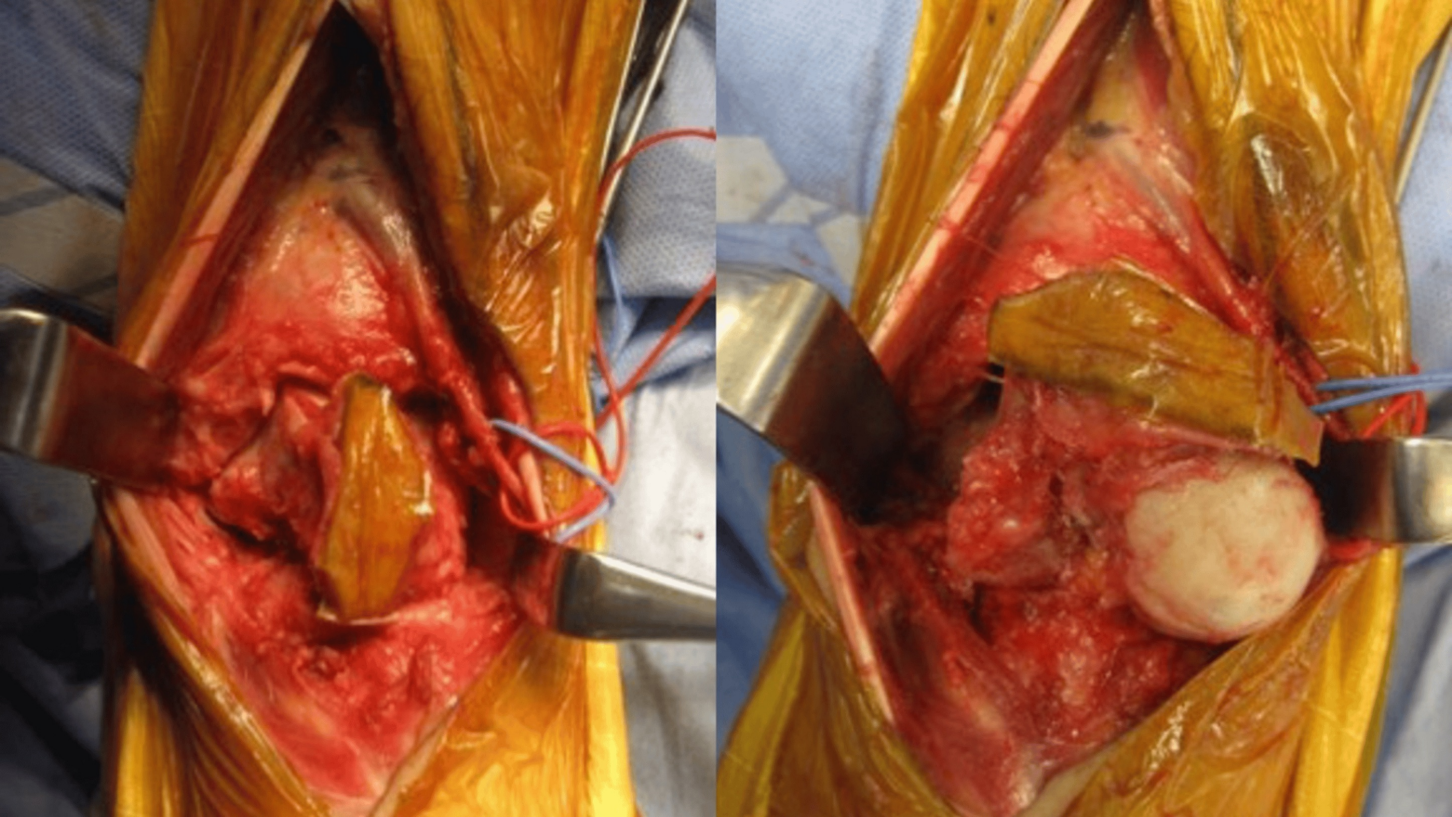
Intraoperative photo An incision was made with an anterior approach to resect with a biopsy track

**Figure 6:**
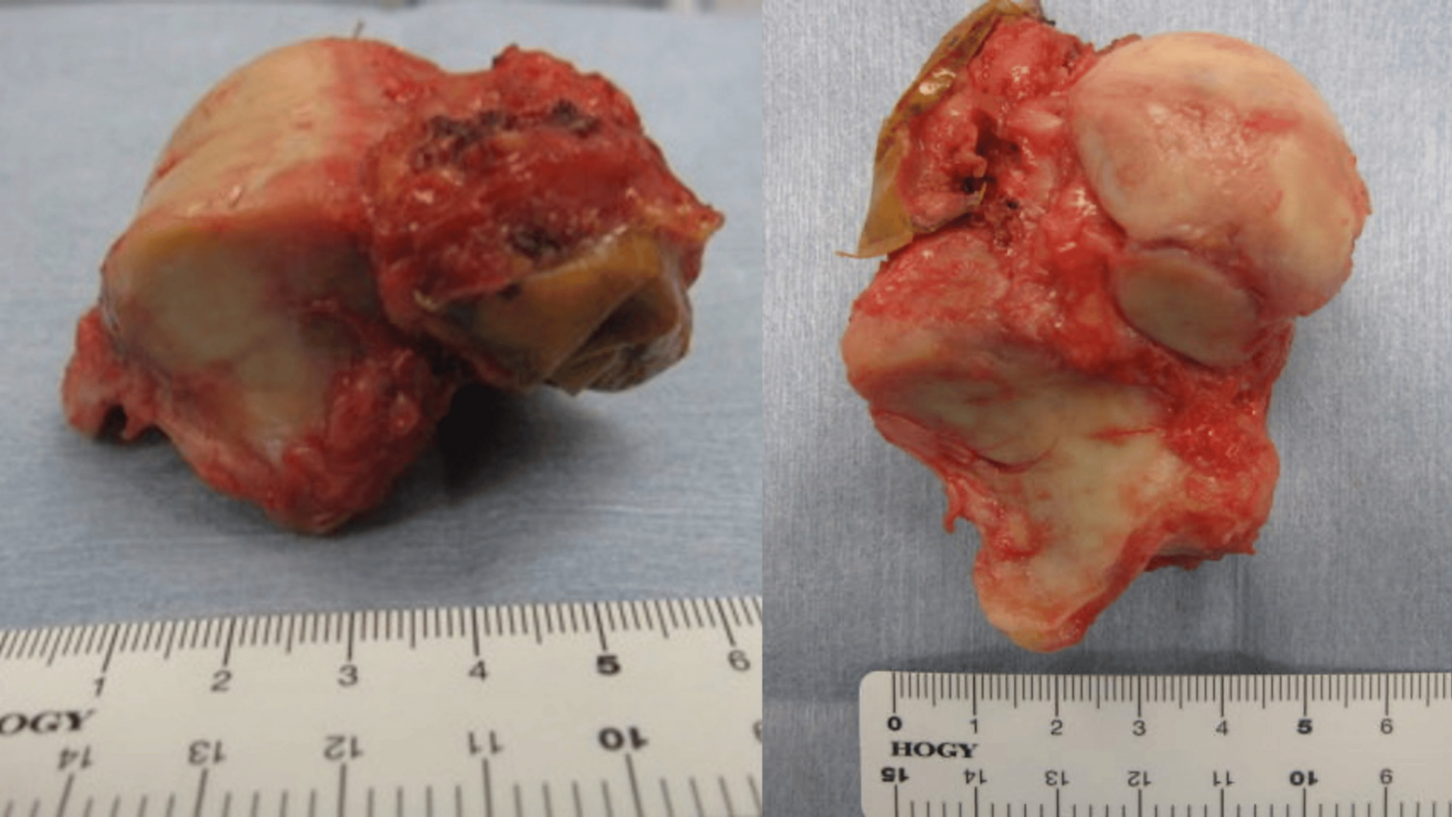
En bloc resection of the talus (lateral and bottom views)

**Figure 7:**
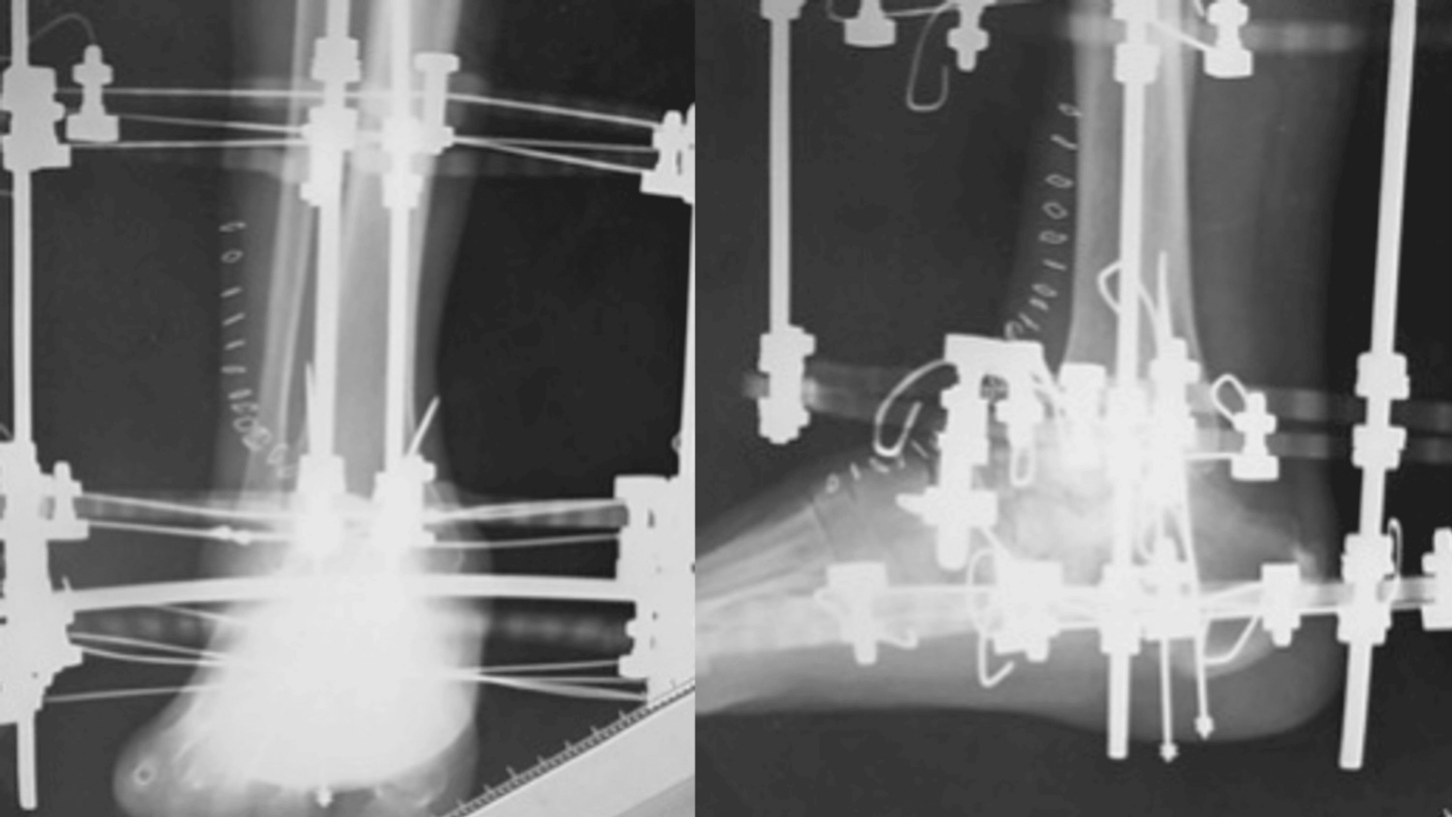
Postoperative radiographs

To achieve wide resection, en bloc resection of the talus was planned. An anterior-approach incision was made to include the biopsy tract. The talonavicular joint capsule was incised, and the subtalar joint was exposed laterally. The deltoid and posterior talotibial ligaments were observed to be tight. After mobilizing the talar head and neck anteriorly, these ligaments were carefully released using Metzenbaum scissors. The talus, including the biopsy tract, was successfully resected (Figures 5-6). Frozen section analysis confirmed no residual tumor in the resected area. Cartilage and the subchondral plate from the tibia and calcaneus were removed using an osteotome and mallet. Bone grafts were harvested from the anterior iliac crest. Tibiocalcaneal arthrodesis was performed using Ilizarov external fixation combined with two trapezoidal strut bone grafts and cancellous bone, as shown in postoperative radiographs (Figure 7). Talonavicular arthrodesis was not performed. The patient was permitted full weight-bearing the day after the surgery.

**Figure 8:**
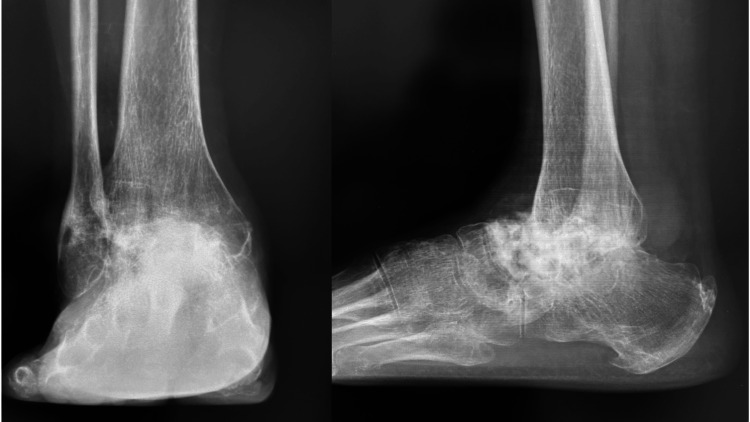
Radiographs at the four-year postoperative follow-up

Chemotherapy was initiated orally two months postoperatively. At 156 days post-surgery, radiographs and CT scans demonstrated successful bony union with good alignment, allowing for the removal of the Ilizarov external fixation device. At the four-year follow-up, the patient remained pain-free, with no recurrence or additional metastases observed (Figure 8). The Japanese Society for Surgery of the Foot (JSSF) ankle/hindfoot scale score was 28 out of 82 points.

## Discussion

Skeletal metastases occur in 20-30% of patients with malignancies [2]. However, acrometastases are exceedingly rare, observed in only 0.007-0.3% of such cases. Previous studies have reported that foot and ankle metastases constitute approximately 2% of all skeletal metastases [2]. Most cases of talar metastases are associated with additional metastatic lesions. Consequently, isolated metastatic lesions of the talus are exceptionally rare. To date, only three cases of isolated talar metastases have been reported in the literature [1,3,4].

When acrometastases in the talus are accompanied by other metastases, primary treatment often includes chemotherapy or radiation therapy tailored to the underlying carcinoma. Chen et al. described multiple bone metastases involving the talus and several vertebrae from advanced renal carcinoma [5]. To alleviate pain, percutaneous osteoplasty was performed, followed by radiation therapy and treatment with zoledronic acid. As for isolated talar metastases, Gan et al. reported treating a talar metastasis from pulmonary carcinoma with lesion curettage and allogeneic bone implantation [3]. Initially misdiagnosed as a benign tumor, malignancy was later confirmed, leading to additional biologic-targeted therapy in combination with zoledronic acid. Similarly, Jaffe et al. reported treating isolated talar metastasis by freezing residual cancer cells with liquid nitrogen after lesion curettage, followed by filling the talar void with polymethyl methacrylate [4]. In advanced carcinoma or cases associated with poor prognoses, palliative care is often prioritized. However, when surgical resection offers a curative approach, it should be considered. In our case, curative treatment was deemed achievable through wide resection of the talus, which was combined with tibiocalcaneal arthrodesis.

Tibiocalcaneal arthrodesis is typically performed using an intramedullary nail (IM) or a locking plate (LP) [6,7]. Previous IM designs often failed to provide sufficient rigidity due to the small size of the calcaneal fragment, allowing only one interlocking screw. Klos et al. reported that improved IM devices, which permit the insertion of two interlocking screws into the calcaneus, provide greater stability for tibiocalcaneal arthrodesis [7]. Furthermore, preserving the talar head and neck is considered critical for achieving stability. Aikawa et al. highlighted the advantages of LP for tibiocalcaneal arthrodesis, noting that LP devices provide rigid fixation by functioning as fixed-angle constructs and enabling the placement of multiple screws into the calcaneus at various angles [6]. Additionally, LP devices use a lateral approach, avoiding the need for additional incisions for IM insertion or bone grafting. However, both IM and LP techniques require extended periods of postoperative non-weight-bearing.

LaPorta et al. reported that combining internal and external fixation for arthrodesis is a viable option [8]. External fixation bridges the arthrodesis site, neutralizes stress within the tarsus, and enhances stability, enabling earlier weight-bearing. In our case, preservation of the talar neck and head was not feasible due to the need for complete talar resection. Furthermore, excision of the anterior biopsy tract precluded the use of a lateral approach for LP, as it would have required additional incisions, increasing surgical invasiveness. Consequently, we opted for tibiocalcaneal arthrodesis with Ilizarov external fixation, which facilitated bone union without recurrence.

## Conclusions

Unlike primary bone tumors, talar metastases warrant thorough evaluation to confirm the absence of recurrence at the primary site and the presence of metastases in other locations. While talar metastases are rare, if curative treatment is feasible, tibiocalcaneal arthrodesis using Ilizarov external fixation after excision may offer a valuable option.

## References

[1] Kouvaris JR, Kouloulias VE, Papacharalampous XN, Koutselini HA, Gennatas CS, Limouris GS, Vlahos LJ (2005). Isolated talus metastasis from breast carcinoma: a case report and review of the literature. Onkologie.

[2] Maheshwari AV, Chiappetta G, Kugler CD, Pitcher JD Jr, Temple HT (2008). Metastatic skeletal disease of the foot: case reports and literature review. Foot Ankle Int.

[3] Gan K, Shen Y (2017). Metastatic pulmonary adenocarcinoma of the talus: a case report. J Foot Ankle Surg.

[4] Jaffe D, Kim E, Aboulafia A (2016). Erosive breast cancer metastasis to the ankle: a case report. J Foot Ankle Surg.

[5] Chen M, Tang H, Feng F (2021). Percutaneous osteoplasty for the management of a talar metastasis: a case report. J Pain Res.

[6] Aikawa T, Watanabe K, Matsubara H, Nomura I, Tsuchiya H (2016). Tibiocalcaneal fusion for Charcot ankle with severe talar body loss: case report and a review of the surgical literature. J Foot Ankle Surg.

[7] Klos K, Drechsel T, Gras F, Beimel C, Tiemann A, Hofmann GO, Mückley T (2009). The use of a retrograde fixed-angle intramedullary nail for tibiocalcaneal arthrodesis after severe loss of the talus. Strategies Trauma Limb Reconstr.

[8] LaPorta GA, Nasser EM, Mulhern JL (2014). Tibiocalcaneal arthrodesis in the high-risk foot. J Foot Ankle Surg.

